# Care-Seeking Patterns and Direct Economic Burden of Injuries in Bangladesh

**DOI:** 10.3390/ijerph14050472

**Published:** 2017-04-29

**Authors:** Yira Natalia Alfonso, Olakunle Alonge, Dewan Md Emdadul Hoque, Md Kamran Ul Baset, Adnan A. Hyder, David Bishai

**Affiliations:** 1 Department of Population Family and Reproductive health, International Injury Research Unit, Johns Hopkins University Bloomberg School of Public Health, Baltimore, MD 21205, USA; dbishai@jhu.edu; 2 Department of International Health, International Injury Research Unit, Johns Hopkins University Bloomberg School of Public Health, Baltimore, MD 21205, USA; oalonge1@jhu.edu (O.A.); ahyder1@jhu.edu (A.A.H.); 3 Maternal and Child Health Division, International Centre for Diarrhoeal Disease Research Bangladesh, Dhaka 1212, Bangladesh; emdad@icddrb.org; 4 Centre for Injury Prevention and Research, Dhaka 1206, Bangladesh; kamran_baset@yahoo.co.uk

**Keywords:** injuries, cost, out-of-pocket, economic burden, care-seeking patterns, low-and-middle-income countries, Bangladesh

## Abstract

This study provides a comprehensive review of the care-seeking patterns and direct economic burden of injuries from the victims’ perspective in rural Bangladesh using a 2013 household survey covering 1.17 million people. Descriptive statistics and bivariate analyses were used to derive rates and test the association between variables. An analytic model was used to estimate total injury out-of-pocket (OOP) payments and a multivariate probit regression model assessed the relationship between financial distress and injury type. Results show non-fatal injuries occur to 1 in 5 people in our sample per year. With average household size of 4.5 in Bangladesh--every household has an injury every year. Most non-fatally injured patients sought healthcare from drug sellers. Less than half of fatal injuries sought healthcare and half of those with care were hospitalized. Average OOP payments varied significantly (range: $8–$830) by injury type and outcome (fatal vs. non-fatal). Total injury OOP expenditure was $355,795 and $5000 for non-fatal and fatal injuries, respectively, per 100,000 people. The majority of household heads with injuries reported financial distress. This study can inform injury prevention advocates on disparities in healthcare usage, OOP costs and financial distress. Reallocation of resources to the most at risk populations can accelerate reduction of preventable injuries and prevent injury related catastrophic payments and impoverishment.

## 1. Introduction

In Bangladesh, the burden of disease from injuries in 2013 was 3031 disability-adjusted-life-years (DALYs) per 100,000 people, which was slightly lower than the global rate, 3456 DALYs per 100,000, and made about 10% of the national disease burden [1]. To prevent injury related disabilities and deaths prompt access to high quality post-injury healthcare is crucial. However, there is increasing evidence across low-and-middle-income-countries (LMIC) that there are major barriers to access health facilities and surgical care [2]. Some of these barriers include long distance to facilities, poor roads and lack of suitable transport, few healthcare providers with resources and expertise, fear of healthcare procedures, high direct and indirect costs related to healthcare as well as socioeconomic status [2]. Barriers to access perpetuate patients’ preferences for self-care, faith on traditional healers (e.g., consultation with Kabiraji/hakimiand homeopathic practitioners), unqualified allopathic providers or para-professionals (village practitioners with a year-long training) [3], and may also reduce completion of healthcare treatments. Further, either high healthcare expenditures or inadequately treated injuries that reduce individual’s work productivity can push families into financial distress (i.e., reduce household income or assets to pay for basic necessities such as food, clothing, housing, school, etc.) worsening the burden from injuries.

Out of the total health expenditure of $16.20 per capita in 2010, 64% was covered out-of-pocket (OOP), 26% by public funds and the remaining by international development partners [4,5]. Between 1997–2007 OOP grew at 14% annually, faster than the annual gross domestic product (GDP), 10% [5]. Similarly, a cross-sectional study from 2009 showed that 94% of injury victims in Bangladesh had an OOP expenditure related to the injury [6]. The growing reliance on OOP expenditures to cover healthcare costs places a large economic burden on individuals needing healthcare services.

When injury related healthcare costs are high compared to family income, payments can absorb a large fraction of household resources resulting in catastrophic expenditures. Injury related expenditures can result from direct medical costs (hospitalization, medicines, labs, etc.), direct non-medical (transportation, food, and accommodation, funeral), indirect costs (income loss due to disability or for caring for a family member with disability), or non-monetary/intangible costs (of premature death, pain and suffering, etc.) [7]. Many south Asian countries like Bangladesh and India have high prevalence of catastrophic payments related to injuries [8,9,10]. 

This study uses 2013 data from a household survey in rural Bangladesh to better understand care-seeking patterns for injuries and the economic burden on victims. The specific aims of this study were to describe care-seeking patterns by injury type, demographic and socioeconomic characteristics; to evaluate injuries’ OOP expenditures by injury type and type of cost; and assess the extent of financial distress due to injury related expenditures. Given the data available, this study evaluates the direct economic burden of injuries from the perspective of the injury victim during the short-term and excludes indirect costs.

## 2. Methods

The data used is from a household survey covering 51 unions (the smallest rural administrative and local government units) from seven sub-districts in rural Bangladesh and was comparable to the 2011 Bangladesh national census [11]. The survey was conducted over a 6-month period (June–November 2013) and covered all 1.17 million individuals in these unions. The survey collected data for all members of a household. Details on the household survey objectives, population, sample design and data management are provided elsewhere [12,13]. This survey was part of the data collected for the Saving of Lives from Drowning (SoLiD) intervention [14,15]. Given that the survey’s recall period for non-fatal injuries was 6 months and for fatal injuries was 1 year, we multiply rates for injuries and cost data for non-fatal injuries by two in order to present all results in person-year units.

Descriptive statistics were used to estimate injury rates and the proportion of each injury out of all injuries by sex, age group and SES (socioeconomic status). Similarly, descriptive statistics were used to estimate the percent of injured persons seeking healthcare by injury type, type of healthcare provider, type of health facility or treatment site, the median number of hospitalization days, and health outcomes (recovered, improving and no improvement). The Chi-squared statistical test was used to assess the association between care provider and injury type as well as, for those hospitalized, injury type and health outcome [16]. 

Descriptive statistics were also used to estimate the proportion of injured individuals with OOP payments greater than zero and the average expenditure among individuals with payments greater than zero by injury type and type of cost (e.g., consultation cost, laboratory cost, bed cost, operation cost, medicine cost, attendant cost, transportation cost, other cost, and total cost). The overall average spending is defined as:

Average Spending = Pr.(Spend > 0) × E(Spend|Spend > 0)(1)

Expenditure data was inflation adjusted from 2013 to 2016 Bangladeshi Taka using inflation index 1.21437 and currency converted to 2016 U.S. dollars using conversion rate of 0.0152. The Wilcoxon-Mann-Whitney statistical test, a non-parametric analog to the independent samples t-test, was used to assess the association between injury type or the probability of getting treatment and OOP expenditures [17,18]. A decision analytic model was developed estimating the total per injury cost for every 100,000 people [7]. This model multiplied the probability of each type of event by the cost of each event. 

Care-seeking patterns and OOP cost estimates were derived separately for non-fatal (injury outcome reported was survived) and fatal (injury outcome reported was death) injuries.

Financial distress was assessed by first estimating the proportion of injured individuals who reported using a financial coping mechanism (e.g., borrow money from relatives/friends, reduced consumption of basic goods, reduced food consumption, took loans, sold assets, other) to cover injury related costs. Then, a probit multivariate regression analysis assessed the association between financial distress (using any financial coping mechanism) and type of injury controlling for demographic and socioeconomic characteristics [19], see Equation (2):

(2) $$\begin{array}{ll} FIN\_Distress_{i}\;= & \beta_{0}+\beta_{1}Injuries_{i}+\beta_{2}Severity_{i}+\beta_{3}Female_{i}+\beta_{4}School_{i}\\ & +\beta_{5}HHsize_{i}+\beta_{6}Age_{i}+\beta_{6}District_{i}\;+\beta_{6}SES_{i}\;+\varepsilon_{i} \end{array}$$

Where *FIN_Distress* is a binomial dependent variable equal to one if the injured person used any financial coping mechanism, $\beta_{0}$ is the constant term, and Injuries is a vector of ten injury dummies where the base case (the least frequent injuries, which made less than 1% of the injury burden and had the lowest total OOP: drowning, suffocation, suicide attempt and unintentional poisoning) is omitted. Thus, the coefficient $\beta_{1}$ is interpreted as the probability of financial distress (multiplied by 100) compared to the base case, holding all other variables constant. Control variables included: Severity, a vector of three dummies where medium and high severity are compared to the omitted dummy of low severity; Female, a dummy for sex; School, a dummy for secondary education; District, a vector of place of residence including Narshindi, Camilla, and Chandpur compared to the omitted base case Sherpur/Siraigonj; SES, a vector of four socioeconomic status with the high income group as the omitted base case; and Age, a vector of four age groups with under-fifteen as the omitted base case. The marginal effect of each coefficient is estimated holding all other coefficients at the mean. The household survey only collected data on use of financial coping mechanism from the injured individuals who were a source of family income (either a minor, main, or major source of family income). Thus, the regression analysis excludes the injuries from individuals who were not contributors to the family income (i.e., children, household keepers, elders or unemployed adults). As such, these estimates likely underestimate the extent of financial distress among all families with an injured family member.

## 3. Results

The study included 1,169,593 individuals from 270,387 households. Details on demographic and injury rates of the study population are provided elsewhere (see Supplementary Materials) [12,13]. Annually there were 238,929 injuries during the year prior to the survey among 209,059 individuals (17.9% of the population). In other words, almost 1 out of every 5 individuals had at least one injury during the year. The total injury rate was 20,428 per 100,000 people. Among the injured, 0.2% (449) died due to the injury. Among those with non-fatal injuries, 11.5% (23,976) and 2.2% (4496) had a second and third injury, respectively. Among non-fatal injuries, only 0.3% of the injury data was missing. The non-fatal injury rate was 20,390 per 100,000 people (238,480 total injuries); falls had the highest rate (7741), followed by cuts (4499) and blunt object injuries (2015). The total fatal-injury rate was 38 per 100,000 people (449 total injuries); the highest was among drowning (15), followed by transport injuries (7) and falls (5).

### 3.1. Healthcare Seeking Patterns and Hospitalization

Overall, 88% of the non-fatal injured sought healthcare treatment; 80% went to a pharmacy/medicine shop, 81% got care from a drug seller/village doctors and 3% were hospitalized (Table 1). The probability of care was not largely different between low, medium and high injury severity levels, ranging from 85% to 94% (see Supplementary Materials). Out of those hospitalized, 34% recovered and 62% were still improving at the time of the survey. Suicides (36%) and poisoning injuries (24%) were most likely to be hospitalized. Among the fatally injured, 45% sought treatment; among the treated, 67% and 48% of healthcare was provided by a doctor and at a hospital, respectively, and 51% were hospitalized.

### 3.2. Out-of-Pocket Expenditures Related to Injury Treatment

**Figure 1:**
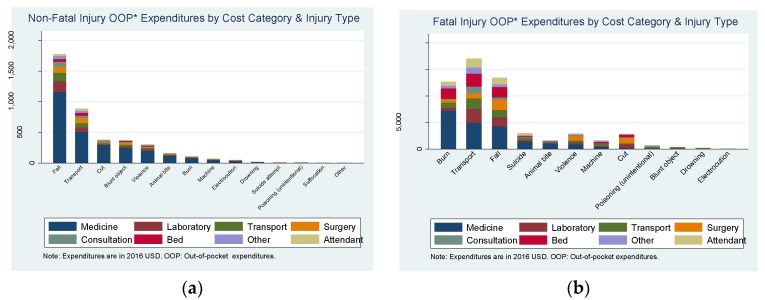
Total direct out-of-pocket expenditure from injuries for every 100,000 people from the study area.

**Figure 2:**
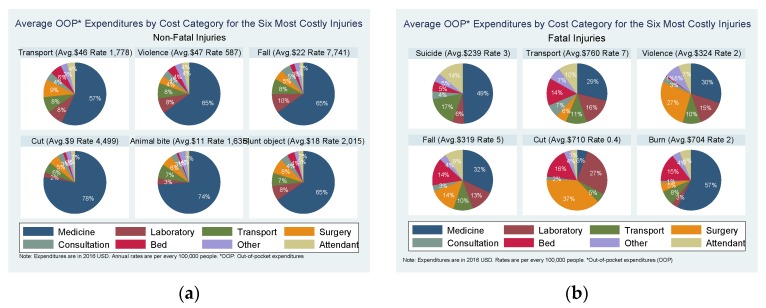
Non-fatal injuries direct average cost by cost category and injury type.

The majority of non-fatal injuries (96%) reported an OOP expense. The Mann-Whitney statistic showed a statistically significant association between OOP costs and getting treatment as well as getting treatment from a registered doctor (*p*-value 0.000). The total annual non-fatal OOP expenditure was $4.16 million (a rate of $355,795 per every 100,000 people) and the average OOP cost (excluding the 4% of injuries with zero expenditure) was $21. The probability of expenditures between the lowest and highest SES only varied from 95% to 97%. Among those with expenditures, the mean expenditure between SES levels varied from between $16 and $26 for the low and high SES groups respectively. Out of the total expenditure, the majority is from falls (43%) and transport injuries (22%) (see Figure 1a). The highest average OOP costs were for suicide attempt ($84), unintentional poisoning ($55), violence ($47) and transport ($46) (see Figure 2a). The probability of OOP payments for medicines was 95%, for transport 31% and for consultation fees 15%. Medicines made 65% of the total OOP cost. Details on costs by injury type and cost category are provided in the Supplementary Materials.

Among fatal injuries, 74% incurred OOP expenses. The total fatal-injury OOP expenditure was $59,672 (a rate of $5114 per 100,000 people) and the average (excluding the 26% of injuries with zero expenditure) was $395 (see Supplementary Materials). Out of the total cost, the majority is from transport injuries (33%), falls (22%), and burn injuries (22%) (see Figure 1b). The highest average OOP costs among fatal injuries were due to machine injuries ($830), transport ($760), cuts ($710) and burns ($704). Similarly, the average cost was highest for surgeries, followed by medicines, and hospital beds (see Supplementary Materials). The average expenditure, for injuries with expenses greater than zero, was 38% of the total GDPpc ($1033) and for the more costly injuries was between 68% (burns) to 80% (machine injuries) of the GDPpc [20]. 

### 3.3. Financial Coping Mechanisms

Among the individuals with non-fatal and fatal injuries, 35% and 27%, respectively, were a source of family income. Most of these individuals were married males, a third had secondary education and 95% where over eighteen years old. Among this group, 90% reported using at least one financial coping mechanism to cover injury related costs. Borrowing money was the most frequent mechanism and more prevalent among fatal injuries than non-fatal injuries (56% vs. 29%). In bivariate analysis, the probability of financial distress was not statistically different by type of injury (see Supplementary Materials). Similarly, multivariate regression analysis, controlling for all injury types, demographic and SES factors, showed no association between financial distress and injury type, expect for a slight positive association between violence or machine injuries (2.8% or 3.3% respectively, *p*-value < 0.05) and financial distress compared to the base case (see Table 2). 

## 4. Discussion

This study evaluated the care-seeking patterns and economic burden of injured individuals in Bangladesh using a household survey from 2013 covering 1.17 million rural people. Overall, non-fatal injuries occur to 1 in 5 people in our sample per year. With average household size of 4.5 in Bangladesh—every household has an injury every year. Highest risk age group is 25 to 64 year olds followed by 5 to 9 year olds. Risk is 1.3 times higher in males than females. Care-seeking patterns show that the probability of seeking treatment is lower for non-fatal than fatal injuries (88% vs. 45% respectively). Among non-fatal injuries, most individuals sought healthcare services from drug sellers/village doctors and at a pharmacy shop. A similar study on burn injuries among 0–18 year olds also found that a significant portion sought care from unqualified service providers (60% vs. 85% and 17% from non- and fatal injuries, respectively) [21]. Among the fatally injured individuals that sought treatment, less than half received care at a hospital. 

Almost all (96%) of non-fatal injuries require out of pocket costs and average $21 and $395 (which is 38% of the Bangladeshi gross domestic product (GDP) per capita, $1033 in 2013) for non- and fatal injuries, respectively. A third of the OOP cost was from medicine expenditures, averaging $13 and $178 for non- and fatal injuries respectively. The low probability for seeking registered doctors and hospitalization may be explained by barriers to healthcare access or OOP costs deterring usage [2,9]. For instance, among non-fatal injuries with OOP expenditures, the average cost for care by a registered doctor was $76 higher than by other healthcare providers, or getting care at a hospital or clinic was $69 higher than getting care at a pharmacy or home. Similarly, among fatal injuries, being hospitalized cost on average $545 more than not being hospitalized. These large direct OOP payments are catastrophic expenditures and can push households into poverty.

The largest portion of OOP expenditure was from falls and transport injures. These injuries were among the costliest because of their high injury rate and average cost. Transport injuries had a hospitalization rate of 7% with a median of 3 hospitalization days. A similar study on transport injuries in Bangladesh found that these injuries were responsible for 5% of hospitalizations in primary and secondary level hospitals, averaged 5.7 hospitalized days and cost on average $86 (in 2010 USD) [22]. Another study on burn injuries found an average cost of $217 (in 2008 USD) compared to $11 and $704 for non- and fatal injuries, respectively, in this study [23]. Drowning, suffocation and electrocution had the lowest average cost because these were the least likely to seek care. But, if indirect costs were included, these fatal injuries would be the costliest due to their lifelong lost productivity/income.

Previous evidence also showed that the majority of injured victims in Bangladesh had OOP expenditures and similarly mostly for medicines, but average medicine OOP cost were lower than in this study ($4 vs. $13) [6]. While this value may seem a small fraction out of the total GDP per capita, this cost can be catastrophic for families in rural areas which has reported suffering from a 36% poverty rate, inadequate diet, periods of food shortage, and having half of the children chronically malnourished [24]. Similarly, among fatal injuries, the average direct OOP cost is 38% of the GDP per capita. 

Reducing the direct economic burden of injured individuals could save Bangladesh $356 thousand plus $5 thousand, from non-fatal and fatal injuries, respectively, for every 100,000 people. These costs only reflect short-term direct savings but are substantially higher if considering the long-term direct OOP costs and indirect costs. Prior evidence from LMICs show that while the median direct medical cost for hospitalization was $291 (range $14–17,400), after adding indirect costs, the median cost increased a 14-fold to US$4085 (range: $17–10,300) or 97% of GDP per capita [25]. While the indirect cost and long-term direct estimates are beyond the scoop of this study, this study’s results show that most household heads with injuries suffered financial distress from injury treatment related costs.

This study was limited by six months (for non-fatal injuries) to one year (for fatal injuries) of self-reported injury and healthcare use and cost data. Also results only captured the short-term effects of injuries. A longer study period could capture the long-term direct costs, particularly for the majority of injuries still in recovery (64%) at the time of the survey. This study also excludes the economic burden from indirect costs which are higher than direct costs [26,27]. Similarly, direct medical costs covered by other public and private financing sources are not included. However, this study’s focus was on capturing the direct OOP expenditures which are often not available in the cost of healthcare studies. Lastly, financial coping mechanisms data was only available for the sub-group of the population with injuries who were a source of family income. Data on financial coping mechanisms from all injured individuals would show the full extent of financial distress among families with injured members. 

## 5. Conclusions

Non-fatal injuries occur to 1 in 5 people in our sample per year. Living in Bangladesh imposes an “injury tax” of $21 per household per year. This does not account for indirect costs, the pain, suffering, and income losses. High risk makes this a prevalent pathway into economic distress. One of the best things Bangladesh can do for its citizens and its economy is to make communities safer. There are promising policies that Bangladeshi communities can use to remove this “injury tax”. Leading economic risks are falls, transport, cuts, blunt objects, violence, animal bites, and burns. Measures include enforce occupational safety regulations, worksite inspection, home safety inspection and promotion, CHW (community health worker) training on safe homes and farms, animal control for stray/wild dogs. 

## Figures and Tables

**Table 1:** Annual care-seeking patterns, hospitalization and health outcome rates of injured persons

Injury Care-Seeking Patterns	Non-Fatal Injuries	Fatal Injuries
(Percent Values Are × 100)		
Annual number of injuries	238,480	449
Percent that sought treatment	0.88	0.45
Out of the total with treatment, percent that received healthcare from each type of provider		
Drug seller/Village doctor	0.81	0.21
Registered doctor	0.14	0.67
Traditional healer/Religious Leader	0.06	0.07
Medical Assistant/SACMO	0.03	0.22
HA/FWV/FWA	0.01	0.02
Other (NGO, homeopathic practitioner, trained TBA)	0.02	0.03
Out of the total with treatment, percent that received healthcare from each type of healthcare facility		
Pharmacy/medicine shop keeper	0.8	0.16
Private (Clinic or practitioner’s chamber)	0.1	0.22
Own home	0.08	0.07
Upazila Health Complex	0.04	0.25
Hospital (District or Specialized)	0.04	0.48
Clinic (NGO or Public Primary Health)	0.01	0.04
UHFWC—Union Health and Family Welfare Centres	0.01	
Other	0.02	
Hospitalization among those treated		
Hospitalizations	0.03	0.51
Median number of hospitalization days	4	4
Treatment outcome among those with hospitalizations		
Recovered	0.34	-
Improving	0.62	-
No improvement	0.04	-

Sub-Assistant Community Medical Officer (SACMO); Health Assistant (HA); Family Welfare Visitor (FWV) or Assistant (FWA). Details on care-seeking patterns by injury type are provided in the Supplementary Materials.

**Table 2:** Regression results on association between financial distress and injury type.

Variables	1	2	3	4	5
	Fin. Stress	Fin. Stress	Fin. Stress	Fin. Stress	Fin. Stress
Injury: Transport	0.023 *	0.020	0.017	0.017	0.018
Injury: Violence	0.037 ***	0.033 **	0.031 **	0.029 **	0.028 **
Injury: Fall	0.025 **	0.024 **	0.026 **	0.018	0.018
Injury: Cut	0.012	0.015	0.015	0.018	0.018
Injury: Burn	0.001	0.005	0.010	0.015	0.016
Injury: Machine	0.038 ***	0.038 ***	0.034**	0.032 **	0.033 **
Injury: Electrocution	0.007	0.008	0.007	0.009	0.010
Injury: Animal Bite	0.000	0.005	0.006	0.009	0.010
Injury: Blunt Object	0.012	0.021 *	0.019	0.013	0.013
Injury severity: Medium		0.031 ***	0.03 ***	0.022 ***	0.022 ***
Injury severity: High		0.022 ***	0.021 ***	0.016 ***	0.016 ***
Family/household size			0.002 ***	0.001 **	0.001 **
Female			−0.014 ***	−0.006 ***	−0.006 ***
Secondary education			0.005 **	0.001	0.002
Age: 15–24 yrs.				0.006	0.005
Age: 25–64 yrs.				0.01 **	0.01 **
Age: 65+ yrs.				0.013 **	0.013 ***
District: Chandpur				0.053 ***	0.053 ***
District: Sherpur				−0.011 ***	−0.011 ***
District: Narshindi				−0.010 ***	−0.010 ***
SES: Medium					0.006 **
SES: Low					0.008 ***
SES: Lowest					0.006 **
Observations	42,327	42,327	42,327	42,327	42,327

*** *p* < 0.01, ** *p* < 0.05, * *p* < 0.1. Data includes the injuries from individuals who were a source of family income (35% of total fatal-injuries). Data excludes fatal injuries. Coefficients are interpreted as percentage point changes × 100 compared to their base case. The base case are the least frequent injuries with lowest total OOP: suffocation, suicide attempt, poisoning and drowning. For example, among this sub-set of the population, there is a 2.8% percentage point increase in the probability of financial distress among violence injuries compared to the base case, holding other factors constant. For details and regression tests see the Supplementary Materials.

## Supplementary Materials

The following are available online at http://www.mdpi.com/1660-4601/14/5/472/s1, Care-Seeking Patterns and Direct Economic Burden of Injuries in Bangladesh.

## Abbreviations

The following abbreviations are used in this manuscript:

OOP	Out-of-pocket
LMIC	Low-and-middle-income-countries
HIC	High-income-countries
NGO	Non-governmental organization
SES	Socioeconomic status
GDP	Gross domestic product
