# Supplementary material for: Care-Seeking Patterns and Direct Economic Burden of Injuries in Bangladesh
Source: Int J Environ Res Public Health. 2017 Apr 29;14(5):472. doi: 10.3390/ijerph14050472 (PMC5451923; doi:10.3390/ijerph14050472)
Supplement: Supplementary file 1 [file ijerph-14-00472-s001.pdf]

Care-Seeking Patterns and Direct Economic Burden of Injuries in Bangladesh

Contents

INTRODUCTION ... 1
PART 1 – SUMMARY OF STUDY POPULATION DEMOGRAPHIC DATA AND INJURY RATES ... 2
Table A1 – Non-fatal injury rates & percent of injuries by injury type, sex, age group & SES ... 3
Table A2 – Fatal injury rates & percent of injuries by injury type, sex, age group & SES ... 3
PART 2 – CARE-SEEKING PATTERNS ... 4
Table B1 – Non-fatal injuries care-seeking patterns by injury type ... 4
Table B2 – Fatal injuries care-seeking patterns by injury type ... 5
PART 3 – OUT-OF-POCKET (OOP) EXPENDITURES ... 6
Table C1 - Non-fatal injuries total, average & probability of direct out-of-pocket (OOP) expenditures (in 2016 USD) by injury and type of cost (proportions are x100) ... 6
Table C2 - Fatal injuries total, average & probability of direct out-of-pocket (OOP) expenditures (in 2016 USD) by injury and type of cost (proportions are x100) ... 7
Table C3 – Non-Fatal injuries out-of-pocket (OOP) expenditures by socioeconomic status ... 8
Table C4 – Fatal injuries out-of-pocket (OOP) expenditures by socioeconomic status ... 8
Table C5 – Non-Fatal injuries out-of-pocket (OOP) expenditures by injury severity ... 9
Table D1 - Non-fatal injuries analytic decision tree model to estimate total injury cost for a hypothetical population of 100,000 people by injury type ... 10
Table D2 - Fatal injuries analytic decision tree model to estimate total injury cost for a hypothetical population of 100,000 people by injury type ... 11
PART 4 – FINANCIAL COPING MECHANISMS ... 12
Table E1 - Non-fatal injuries probability of financial distress & coping mechanisms by injury type ... 12
Table E2 - Fatal injuries probability of financial distress & coping mechanisms by injury type ... 12
Table F1 – Descriptive statistic for variables included in the regression models ... 13
Table F2 – Probit regression models ... 14

INTRODUCTION

This appendix provides detail data on the care-seeking patterns of injured persons, their out-of-pocket (OOP) expenditures and financial coping mechanisms. Part 1 lists injury rates by injury type, demographic and socioeconomic (SES) factors. Part 2 lists the probability of seeking health care by injury type, demographic and SES factors. Part 3 shows details on the probability of OOP expenditures, average and mean cost by injury type and type of cost and illustrates the analytic decision tree model used to estimate total injury OOP cost per injury for a hypothetical population of 100,000 people. Lastly, part 4 presents detail results on the proportion of injury victims using various types of financial coping mechanisms as well as regression results on the association between financial distress and injury type.

PART 1 – SUMMARY OF STUDY POPULATION DEMOGRAPHIC DATA AND INJURY RATES

Details on the demographic and injury rates of the study population are provided elsewhere[1]. However, the data below provides a summary of main demographic and injury rate characteristics important for understanding the distribution of injury related out-of-pocket (OOP) expenditures and the financial burden on injured persons.

The study included 1,169,593 individuals from 270,387 households. Among the study population 51% were female. Upazila of residence was 23% Matlab North, 19% Sherpur, 18% Matlab South, 17% Manohardi, 11% Chandpur Sadar, 9% Raiganj and 2% Daud Kandi. The age distribution was 34% less than 15, 17% 15-24, 44% 25-64 and 6% 65 and up. While 49% were married and 5% widowed, 19% never married. Among the 18-64 year olds the work activities were 53% housewife or unemployed/retired (of which 82% were married females) and 14% agriculture, 13% and 9% professional skilled labor and business, respectively. Among the 18-64 years old, 31% had no education, 29% and 31% completed primary and secondary school, respectively.

Results below describe non-fatal and fatal injury rates separately. The study collected data on non-fatal injuries from the six months prior to the survey so we multiplied injuries by two in order to report rates in person-years as it is commonly done by epidemiologists. The non-fatal injury rate was 20,390 per 100,000 people. See Table A1 for details. Among all injuries, falls had the highest rate (7,741), followed by cuts (4,499), blunt object injuries (2,015), transport (1,778), animal bites (1,635) and others. Falls also had the highest injury rates among females and males, all age groups and SES. Comparing the frequency of injuries by sex shows that, while falls were more frequent in females (49%) than males (29%), cuts were more frequent in males (25%) than females (18%). Injuries by blunt objects and transport were also more frequent in males than females but burns were more frequent in females than males (and the third most frequent injury in females). Similarly, while falls were the most common injury among all age groups, cuts were the second most frequent among the 5-54 year olds, and drowning was the second most frequent among 1-4 year olds. Injury by blunt object and transport were the third and fourth most frequent, respectively, among the 15-24-year-old males. Injury rates did not vary much between the five different socioeconomic statuses.

Fatal injury rates (38 per 100,000 people) were highest for drowning (15), followed by transport injuries (7), falls (5), suicide (3), burns (2) and others, see Table Ab. Rates were similar for both males and females with higher drowning and transport injury rates for males. Fatal drowning was highest among 1 to 4 year olds at 110/100,000. Fatal transport injuries were most frequent among the 25-64 year olds and fatal fall injures were most frequent among 65+ year olds. Suicide was highest among 15-17 year olds at 11/100,000. Suffocation, drowning and cut injuries death rates were highest among children less than one-year-old with death rates of 14, 9 and 9 respectively.

---

[1] Source 1: Alonge, O., et al., Fatal and non-fatal injury outcomes in Bangladesh: Results from a large population study. This manuscript is currently under peer review, see summary of selected methods below. Source 2: He, S.; Alonge, O.; Agrawal, P.; Sharmin, S.; Islam, I.; Mashreky, S.R.; Arifeen, S.E. Epidemiology of Burns in Rural Bangladesh: An Update. Int. J. Environ. Res. Public Health 2017, 14, 381.

"A baseline census was conducted in 2013 as part of an injury prevention intervention study, "Saving of Lives from Drowning (SoLiD)" in seven sub-districts (Upazilas) of rural Bangladesh to establish epidemiological characteristics of fatal and non-fatal injuries. The sub-districts were purposively selected because of their higher risk for childhood drowning. The study was implemented with support from two local organizations, Center for Injury Prevention and Research, Bangladesh (CIRPB) and International Center for Diarrheal Disease Research, Bangladesh (icddr,b). The seven selected sub-districts were Raiganj, Sherpur, Manohardi, Matlab South, Matlab North, Daudkandi, and Chandpur Sadar. The census covered 993 villages, 270,387 households and a population of approximately 1.2 million in 51 Unions of the seven selected sub-districts. Household members were interviewed on a one-on-one basis (typically one member responded on behalf of one household) to retrieve required information using questionnaires developed and tested for the SoLiD study. Data collection was done in two stages. The first round collected general demographic information on all members of a household as well as any record of injury in the past six months and deaths in the past one year. If an individual reported a particular injury mortality or morbidity event during the first round of data collection, an injury specific form was used to obtain detailed information about the injury and death in a second round of data collection. Injury was defined as any external harm resulting from an assault, fall, cut, burn, animal bite, poisoning, transportation of goods and persons, operating machinery, blunt objects, suffocation, and (near) drowning resulting in the loss of one or more days of normal daily activities, schools, or work."

Table A1 – Non-fatal injury rates & percent of injuries by injury type, sex, age group & SES

External cause of injury (values are person-year rates per every 100,000 people)	Fall	Cut	Blunt object	Transport	Animal bite	Burn	Drowning	Violence	Machine	Electrocu-tion	Suffoca-tion	Poisoning (Uninten-tional)	Suicide attempt	Others	All Injuries
Rate	7,741	4,499	2,015	1,778	1,635	1,068	642	587	220	146	33	15	10	0	20,390
Number of non-fatal injuries	90,544	52,622	23,572	20,796	19,126	12,486	7,504	6,860	2,578	1,710	384	174	120	4	238,480
Percent out of total injuries	38	22	10	9	8	5	3	3	1	1	0.2	0.1	0.1	0	100
MALE	6,791	5,853	2,939	3,093	1,831	636	750	745	399	176	30	15	10	0	23,268
FEMALE	8,643	3,214	1,139	530	1,449	1,477	539	436	51	118	35	15	11	0	17,658
AGE GRP. <1yr	2,882	638	219	128	182	566	337	9	0	0	9	0	0	0	4,971
AGE GRP. 1-4yr	6,919	3,699	1,210	750	851	2,518	6,169	238	73	90	26	66	2	0	22,611
AGE GRP. 5-9yr	6,716	4,465	1,460	1,445	1,738	828	943	449	92	111	19	11	3	0	18,279
AGE GRP. 10-14yr	5,703	4,211	1,852	1,482	1,590	537	75	435	95	139	11	4	6	0	16,140
AGE GRP. 15-17yr	4,912	3,793	2,151	2,144	915	686	29	527	0	158	26	223	13	0	15,578
AGE GRP. 18-24yr	4,593	3,602	1,907	2,026	946	1,075	39	549	290	161	17	6	20	0	15,231
AGE GRP. 25-64yr	9,410	5,361	2,494	2,162	2,086	1,171	57	794	323	174	42	15	13	1	24,102
AGE GRP. 65+	12,720	3,471	1,673	1,309	1,656	470	53	420	115	112	90	14	6	0	22,107
SES - LOWEST	7,805	5,095	2,020	1,754	1,918	1,211	1,021	774	214	77	33	17	10	0	21,951
SES - LOW	7,770	5,173	2,110	1,841	1,961	1,087	760	665	253	100	38	17	13	0	21,789
SES - MIDDLE	8,210	4,605	2,175	1,633	1,565	1,027	603	604	255	154	27	14	12	0	20,885
SES - HIGH	7,863	4,436	2,166	1,730	1,577	1,039	513	550	231	183	41	11	9	1	20,350
SES - HIGHTEST	7,292	3,488	1,680	1,972	1,280	1,024	400	395	160	204	27	15	8	1	17,944

Table A2 – Fatal injury rates & percent of injuries by injury type, sex, age group & SES

External cause of injury (values are rates per every 100,000 people)	Drowning	Transport	Fall	Suicide	Burn	Violence	Electrocu-tion	Suffoca-tion	Blunt object	Animal bite	Poisoning (unintenti onal)	Cut	Machine	All Injuries
Rate	15	7	5	3	2	2	1	1	1	1	1	0.4	0.3	38
ALL Number of fatal injuries	172	80	59	39	25	20	13	10	9	8	6	5	3	449
Percent out of total injuries	38	18	13	9	6	4	3	2	2	2	1	1	1	100
MALE	17	9	5	4	0	3	2	1	1	1	1	1	0	44
FEMALE	12	5	5	3	4	1	0	1	1	0	0	0	0	33
AGE GRP. <1yr	9	0	0	0	0	0	0	14	0	0	0	9	0	32
AGE GRP. 1-4yr	110	5	0	1	0	0	0	1	0	1	1	0	0	120
AGE GRP. 5-9yr	22	6	2	0	0	0	1	0	1	1	0	0	0	33
AGE GRP. 10-14yr	4	4	1	4	1	1	1	1	0	1	0	1	0	17
AGE GRP. 15-17yr	3	8	0	11	2	2	2	0	2	0	2	2	2	34
AGE GRP. 18-24yr	7	7	2	8	3	2	2	0	0	0	1	0	0	29
AGE GRP. 25-64yr	3	7	3	3	1	3	1	1	1	0	0	0	0	24
AGE GRP. 65+	8	14	55	3	18	3	3	1	1	3	3	0	0	112
SES - LOWEST	20	9	3	3	2	3	2	0	1	1	0	0	0	46
SES - LOW	18	6	5	3	4	1	1	0	0	1	0	0	0	40
SES - MIDDLE	14	6	5	4	3	2	1	0	1	0	1	1	1	38
SES - HIGH	11	9	6	4	1	2	1	1	1	0	1	0	0	38
SES - HIGHTEST	12	5	6	3	1	1	0	2	1	1	0	0	0	32

PART 2 – CARE-SEEKING PATTERNS

Table B1 – Non-fatal injuries care-seeking patterns by injury type

External cause of injury	Fall	Cut	Blunt object	Transport	Animal bite	Burn	Drowning	Violence	Machine	Electrocu-tion	Suffoca-tion	Poisoning (Unintentional)	Suicide attempt	Others	All Injuries
Number of non-fatal injuries	90,544	52,622	23,572	20,796	19,126	12,486	7,504	6,860	2,578	1,710	384	174	120	4	**238,480**
Percent of the injured persons that sought treatment	0.92	0.86	0.91	0.96	0.84	0.85	0.39	0.95	0.96	0.78	0.62	0.87	0.88	1.00	**0.88**
**Out of the total injured persons that sought treatment, percent that received healthcare from each type of provider**															
Drug seller/Village doctor	0.80	0.89	0.86	0.74	0.72	0.85	0.65	0.67	0.81	0.72	0.50	0.51	0.47	0.50	**0.81**
Registered doctor	0.16	0.07	0.12	0.24	0.09	0.07	0.09	0.30	0.18	0.18	0.19	0.45	0.36	1.00	**0.14**
Traditional healer/Religious	0.10	0.00	0.05	0.02	0.09	0.05	0.16	0.02	0.01	0.02	0.27	0.05	0.02	0.00	**0.06**
Medical Assistant/SACMO	0.03	0.02	0.03	0.06	0.02	0.02	0.02	0.08	0.04	0.05	0.07	0.12	0.21	1.00	**0.03**
HA/FWV/FWA	0.01	0.01	0.01	0.01	0.01	0.02	0.01	0.02	0.01	0.02	0.02	0.01	0.02	0.50	**0.01**
Other(NGO, homeopathic practtnr., trained TBA)	0.02	0.02	0.02	0.02	0.04	0.03	0.04	0.01	0.02	0.04	0.10	0.05	0.02	0.00	**0.02**
**Out of the total injured persons that sought treatment, percent that received healthcare from each type of healthcare facility**															
Pharmacy/medicine shop	0.79	0.88	0.84	0.73	0.73	0.85	0.66	0.66	0.79	0.71	0.61	0.53	0.42	0.50	**0.80**
Private (Clinic or practitioner's	0.13	0.04	0.10	0.15	0.05	0.05	0.06	0.15	0.12	0.11	0.18	0.14	0.15	0.50	**0.10**
Own home	0.10	0.05	0.06	0.04	0.13	0.08	0.21	0.04	0.02	0.08	0.23	0.11	0.02	0.00	**0.08**
Upazila Health Complex	0.03	0.03	0.03	0.07	0.03	0.02	0.02	0.13	0.03	0.08	0.05	0.16	0.28	0.50	**0.04**
Hospital (District or	0.04	0.02	0.03	0.08	0.03	0.02	0.02	0.12	0.06	0.07	0.05	0.21	0.23	0.00	**0.04**
Clinic (NGO or Public Primary	0.01	0.01	0.01	0.01	0.01	0.02	0.02	0.01	0.01	0.01	0.00	0.03	0.02	0.00	**0.01**
UHFWC - Union Health and	0.01	0.01	0.01	0.01	0.01	0.01	0.01	0.01	0.01	0.02	0.01	0.01	0.02	0.50	**0.01**
Other	0.04	0.00	0.01	0.01	0.07	0.01	0.06	0.01	0.01	0.00	0.03	0.00	0.00	0.00	**0.02**
**Hospitalization among those treated**															
Hospitalizations	0.02	0.01	0.01	0.07	0.01	0.01	0.02	0.18	0.04	0.07	0.03	0.24	0.36	0.00	**0.03**
Median number of	4	5	12	2	11	6	7	3	9	10	13	8	1		**4**
**Treatment outcome among those with hospitalizations**															
Recovered	0.29	0.43	0.34	0.31	0.42	0.37	0.55	0.35	0.34	0.43	0.67	0.50	0.42	0.00	**0.34**
Improving	0.65	0.54	0.59	0.64	0.56	0.58	0.45	0.62	0.62	0.53	0.33	0.50	0.58	0.00	**0.62**
No improv	0.05	0.02	0.07	0.05	0.03	0.05	0.00	0.03	0.04	0.04	0.00	0.00	0.00	0.00	**0.04**

Table B2 – Fatal injuries care-seeking patterns by injury type

External cause of injury	Drowning	Transport	Fall	Suicide	Burn	Violence	Electrocu-tion	Suffoca-tion	Blunt object	Animal bite	Poisoning (unintentional)	Cut	Machine	All Injuries
Number of fatal injuries	172	80	59	39	25	20	13	10	9	8	6	5	3	**449**
Percent of the injured persons that sought treatment	0.23	0.45	0.86	0.38	0.96	0.50	0.38	0.00	0.56	0.88	0.67	1.00	0.67	**0.45**
**Out of the total injured persons that sought treatment, percent that received healthcare from each type of provider**														
Registered doctor	0.64	0.72	0.65	0.67	0.67	0.60	0.80	0.00	0.40	0.71	1.00	0.60	0.50	**0.67**
Medical Assistant/SACMO	0.13	0.19	0.25	0.13	0.21	0.40	0.20	0.00	0.60	0.43	0.00	0.00	0.50	**0.22**
Drug seller/Village doctors	0.36	0.08	0.29	0.20	0.17	0.10	0.00	0.00	0.00	0.14	0.00	0.40	0.00	**0.21**
Herbal Medicine Practitioner	0.03	0.00	0.20	0.00	0.00	0.00	0.00	0.00	0.00	0.14	0.00	0.00	0.00	**0.06**
HA/FWV/FWA	0.00	0.03	0.00	0.13	0.04	0.10	0.00	0.00	0.00	0.00	0.00	0.00	0.00	**0.02**
NGO service provider	0.03	0.00	0.04	0.00	0.04	0.00	0.00	0.00	0.00	0.00	0.00	0.00	0.00	**0.02**
Traditional healer/Religious Leader/Relatives	0.00	0.00	0.04	0.00	0.00	0.00	0.00	0.00	0.00	0.16	0.00	0.00	0.00	**0.01**
Others (Trained TBA, Homeophatic prict., Other)	0.03	0.03	0.00	0.00	0.00	0.00	0.00	0.00	0.00	0.00	0.00	0.00	0.00	**0.01**
**Out of the total injured persons that sought treatment, percent that received healthcare from each type of healthcare facility**														
Hospital (District or Specialized)	0.15	0.69	0.41	0.47	0.46	0.90	0.80	0.00	0.80	0.71	0.75	0.00	1.00	**0.48**
Upazila Health Complex	0.33	0.14	0.18	0.53	0.21	0.10	0.20	0.00	0.20	0.57	0.50	0.20	0.00	**0.25**
Private Clinic	0.13	0.11	0.31	0.07	0.17	0.10	0.00	0.00	0.20	0.00	0.25	0.40	0.00	**0.17**
Pharmacy	0.28	0.00	0.29	0.00	0.13	0.10	0.00	0.00	0.20	0.00	0.00	0.20	0.00	**0.16**
Own home	0.08	0.03	0.16	0.07	0.08	0.00	0.00	0.00	0.00	0.00	0.00	0.00	0.00	**0.07**
Private chamber	0.13	0.00	0.08	0.00	0.00	0.00	0.00	0.00	0.00	0.00	0.00	0.00	0.00	**0.04**
Others (UHFWC, NGO clinic, other)	0.05	0.03	0.04	0.00	0.04	0.10	0.00	0.00	0.00	0.29	0.00	0.00	0.00	**0.04**
**Hospitalization among those treated**														
Percent of hospitalizations	0.05	0.78	0.39	0.73	0.79	0.70	0.40	0.00	0.60	0.57	0.75	0.60	1.00	**0.51**
Median number of hospitalization days	0	3	7	1	8	4	1	0	3	5	1	15	9	**4**

PART 3 – OUT-OF-POCKET (OOP) EXPENDITURES

Table C1 - Non-fatal injuries total, average & probability of direct out-of-pocket (OOP) expenditures (in 2016 USD) by injury and type of cost (proportions are x100)

External cause of injury	Fall	Cut	Blunt object	Transport	Animal bite	Burn	Drowning	Violence	Machine	Electrocu-tion	Suffoca-tion	Poisoning (Unintentional)	Suicide attempt	Other injuries	All Injuries	Total sum percent distribution
**Total Cost**																
Percent	0.97	0.97	0.97	0.98	0.90	0.95	0.96	0.98	0.78	0.94	0.79	0.95	0.98	1.00	**0.96**	
Sum ($)	1,786,292	379,370	373,706	895,490	156,336	105,598	17,128	303,762	69,666	47,618	8,698	7,978	5,146	372	4,157,162	**1.00**
Injury cost proportion out of the all injuries cost	0.43	0.09	0.09	0.22	0.04	0.03	0.00	0.07	0.02	0.01	0.00	0.00	0.00	0.00	1.00	
Average ($)	22	9	18	46	11	11	8	47	29	38	27	55	84	93	**21**	
Median ($)	5	3	4	8	2	3	3	11	7	5	5	10	34	93	4	
**Consultation Cost**																
Percent	0.18	0.08	0.13	0.23	0.09	0.08	0.18	0.26	0.09	0.18	0.17	0.32	0.32	0.50	**0.15**	
Sum ($)	88,526	11,992	14,910	35,640	4,848	3,640	844	11,040	2,434	1,544	282	402	268	24	176,396	**0.04**
Average ($)	6	3	5	8	4	4	3	7	6	7	7	8	8	12	**6**	
Median ($)	5	2	3	5	3	2	2	5	3	3	6	5	8	12	3	
**Laboratory Cost**																
Percent	0.14	0.01	0.07	0.14	0.02	0.01	0.08	0.18	0.02	0.06	0.13	0.20	0.19	0.50	**0.09**	
Sum ($)	171,382	8,786	28,418	74,082	5,234	1,924	1,220	23,754	3,384	4,604	400	642	572	102	324,506	**0.08**
Average ($)	15	17	19	26	14	24	27	20	18	54	18	21	20	51	**18**	
Median ($)	8	8	9	11	9	14	11	9	11	15	10	11	15	51	9	
**Bed Cost**																
Percent	0.01	0.00	0.01	0.04	0.00	0.01	0.02	0.05	0.00	0.04	0.03	0.03	0.08	0	**0.01**	
Sum ($)	41,288	5,110	14,434	52,030	1,644	3,734	544	12,988	2,660	2,850	414	110	152	-	137,954	**0.03**
Average ($)	55	35	78	74	27	58	54	41	46	51	25	27	52	0	**58**	
Median ($)	23	9	20	23	9	23	15	15	15	15	12	27	23	0	18	
**Operation Cost**																
Percent	0.02	0.02	0.02	0.04	0.01	0.01	0.04	0.04	0.00	0.01	0.01	0.00	0.06	0	**0.02**	
Sum ($)	96,428	19,288	28,768	83,208	9,020	3,432	18	12,218	5,150	4,238	82	-	92	-	261,940	**0.06**
Average ($)	76	21	62	110	69	55	2	43	59	235	46		14	0	**66**	
Median ($)	15	3	8	15	8	3	2	8	14	46	46		9	0	8	
**Medicine Cost**																
Percent	0.96	0.96	0.96	0.97	0.88	0.94	0.96	0.97	0.76	0.93	0.76	0.92	0.98	1.00	**0.95**	
Sum ($)	1,156,940	293,906	243,676	502,952	114,718	80,020	12,194	195,890	45,918	27,784	6,100	5,198	3,240	198	2,688,734	**0.65**
Average ($)	14	7	12	26	8	8	5	31	19	23	18	37	59	49	**13**	
Median ($)	5	2	4	8	2	2	3	9	6	5	5	8	19	49	4	
**Attendant Cost**																
Percent	0.02	0.02	0.02	0.07	0.01	0.02	0.05	0.11	0.02	0.06	0.02	0.20	0.19	0	**0.03**	
Sum ($)	32,784	7,192	9,576	34,908	2,556	3,274	298	11,226	1,980	1,814	352	484	334	-	106,776	**0.03**
Average ($)	16	10	19	25	11	16	6	15	17	24	84	16	18	0	**17**	
Median ($)	6	3	8	8	3	5	3	8	8	8	84	9	8	0	8	
**Transport Cost**																
Percent	0.35	0.23	0.28	0.41	0.21	0.23	0.36	0.47	0.21	0.35	0.30	0.54	0.60	0.50	**0.31**	
Sum ($)	141,930	21,544	25,240	70,888	11,634	6,600	1,596	24,316	5,592	3,360	818	890	400	48	314,854	**0.08**
Average ($)	5	2	4	9	3	3	3	8	6	7	6	11	13	24	**5**	
Median ($)	2	1	2	2	1	1	1	3	2	2	3	5	8	24	2	
**Other Cost**																
Percent	0.09	0.06	0.07	0.15	0.07	0.07	0.12	0.17	0.07	0.14	0.09	0.21	0.34	0	**0.09**	
Sum ($)	47,996	10,428	7,152	30,448	5,802	2,782	414	11,288	2,326	1,424	222	256	90	-	120,628	**0.03**
Average ($)	7	4	5	10	5	4	2	10	8	8	4	8	6	0	**6**	
Median ($)	2	1	2	3	2	1	2	3	2	2	3	6	4	0	2	

Note: *Average cost estimates are based on the average among the individuals with expenditure greater than zero (excludes the individuals with expenditure equal to zero)

Table C2 - Fatal injuries total, average & probability of direct out-of-pocket (OOP) expenditures (in 2016 USD) by injury and type of cost (proportions are x100)

External cause of injury	Drowning	Transport	Fall	Suicide	Burn	Violence	Electrocu-tion	Suffoca-tion	Blunt object	Animal bite	Poisoning (unintentional)	Cut	Machine	All Injuries	Total sum percent distribution
**Total Cost**															
Percent	0.59	0.72	0.82	0.80	0.75	0.90	0.40	0.00	1.00	0.71	0.75	0.80	1.00	**0.74**	
Sum ($)	227	19,749	13,407	2,871	12,674	2,912	17	0	1,018	1,638	658	2,839	1,660	**59,672**	**1.00**
Injury cost proportion out of the all injuries cost	0.00	0.33	0.22	0.05	0.21	0.05	0.00	0.00	0.02	0.03	0.01	0.05	0.03	**1.00**	
Average ($)	10	760	319	239	704	324	9	0	204	328	219	710	830	**395**	
Median ($)	8	205	142	50	536	114	9	0	108	213	144	49	830	**122**	
**Consultation**															
Percent	0.13	0.42	0.53	0.20	0.46	0.50	0.00	0.00	1.00	0.57	0.25	0.40	1.00	**0.39**	
Sum ($)	23	1,180	429	125	123	89	0	0	49	84	8	49	81	**2,237**	**0.04**
Average ($)	5	79	16	42	11	18	0	0	10	21	8	24	40	**28**	
Median ($)	3	15	9	46	8	3	0	0	11	23	8	24	40	**8**	
**Laboratory**															
Percent	0.03	0.33	0.57	0.20	0.29	0.50	0.00	0.00	0.40	0.43	0.25	0.40	1.00	**0.33**	
Sum ($)	30	2,669	1,679	185	441	424	0	0	20	106	12	769	164	**6,500**	**0.11**
Average ($)	30	222	58	62	63	85	0	0	10	35	12	385	82	**97**	
Median ($)	30	62	30	30	30	15	0	0	10	30	12	385	82	**30**	
**Bed**															
Percent	0.00	0.22	0.24	0.20	0.38	0.30	0.00	0.00	0.40	0.14	0.25	0.20	1.00	**0.21**	
Sum ($)	0	2,464	1,940	155	1,914	23	0	0	52	30	30	456	305	**7,370**	**0.12**
Average ($)	0	308	162	52	213	8	0	0	26	30	30	456	153	**175**	
Median ($)	0	266	76	15	137	8	0	0	26	30	30	456	153	**61**	
**Operation**															
Percent	0.00	0.08	0.12	0.00	0.13	0.30	0.00	0.00	0.00	0.00	0.25	0.20	0.00	**0.08**	
Sum ($)	0	987	1,907	0	608	779	0	0	0	0	30	1,063	0	**5,374**	**0.09**
Average ($)	0	329	318	0	203	260	0	0	0	0	30	1,063	0	**316**	
Median ($)	0	152	289	0	228	18	0	0	0	0	30	1,063	0	**152**	
**Medicine**															
Percent	0.08	0.56	0.80	0.67	0.75	0.80	0.00	0.00	0.80	0.71	0.75	0.40	1.00	**0.57**	
Sum ($)	25	4,927	4,283	1,446	7,269	878	0	0	96	995	109	163	425	**20,617**	**0.35**
Average ($)	8	246	104	145	404	110	0	0	24	199	36	81	213	**178**	
Median ($)	9	114	61	45	334	53	0	0	27	99	46	81	213	**76**	
**Attendant**															
Percent	0.08	0.42	0.37	0.27	0.71	0.60	0.00	0.00	0.40	0.43	0.25	0.60	0.50	**0.36**	
Sum ($)	23	1,701	1,264	404	776	199	0	0	21	76	8	91	152	**4,715**	**0.08**
Average ($)	8	113	67	101	46	33	0	0	11	25	8	30	152	**64**	
Median ($)	8	23	15	125	23	11	0	0	11	30	8	12	152	**15**	
**Transport**															
Percent	0.49	0.67	0.63	0.80	0.75	0.80	0.40	0.00	1.00	0.71	0.75	0.80	1.00	**0.66**	
Sum ($)	107	1,934	1,407	486	1,012	288	17	0	117	269	289	142	380	**6,448**	**0.11**
Average ($)	6	81	44	41	56	36	9	0	23	54	96	36	190	**48**	
Median ($)	6	34	15	15	17	11	9	0	15	23	61	33	190	**15**	
**Other Cost**															
Percent	0.10	0.50	0.43	0.33	0.50	0.40	0.00	0.00	0.40	0.57	0.50	0.20	1.00	**0.37**	
Sum ($)	19	1,152	498	138	531	232	0	0	8	77	172	106	153	**3,088**	**0.05**
Average ($)	5	64	23	28	44	58	0	0	4	19	86	106	77	**41**	
Median ($)	5	27	11	8	50	36	0	0	4	21	86	106	77	**15**	

Note: *Average cost estimates are based on the average among the individuals with the expenditure greater than zero (excludes the individuals with the injury but expenditure equal to zero)

The probability of expenditures between the lowest and highest SES only ranges from 95% to 97%. Among those with expenditures, the mean expenditure varies between $16 and 26$ for the low and high SES groups respectively. Similarly, among fatal injuries the probability of expenditure did not vary significantly by SES level but the mean the expenditure did range between $274 to $767. Even the low SES mean cost for fatal injuries is high and may help explain partially the low rate of care-seeking among fatal injuries (203 out of 449).

Table C3 – Non-Fatal injuries out-of-pocket (OOP) expenditures by socioeconomic status

**Cost of non-fatal injuries by SES**	**Fatal injuries by SES**		**Fatal injuries that sought treatment by SES**			**Fatal injuries with expenditure greater than zero (Exp. >0)**				
**SES Index**	**No.**	**% (x100)**	**No.**	**% (x100)**	**Prob. of Exp.>0**	**No.**	**Mean**	**Median**	**Max.**	**Min.**
Lowest	46,218	0.19	40,188	0.19	0.95	38,174	$16	$3	$5,917	$0.03
Low	47,474	0.20	41,850	0.20	0.96	40,120	$19	$4	$4,801	$0.02
Middle	49,556	0.21	44,116	0.21	0.97	42,660	$19	$4	$3,494	$0.03
High	50,142	0.21	44,220	0.21	0.97	42,692	$22	$5	$2,947	$0.03
Highest	45,090	0.19	39,782	0.19	0.97	38,558	$26	$5	$5,498	$0.03
Total	238,480	1.00	210,156	1.00	0.96	202,204	$21	$4	$5,917	$0.02

Table C4 – Fatal injuries out-of-pocket (OOP) expenditures by socioeconomic status

**Cost of fatal injuries by SES**	**Fatal injuries by SES**		**Fatal injuries that sought treatment by SES**			**Fatal injuries with expenditure greater than zero (Exp. >0)**				
**SES Index**	**No.**	**% x100**	**No.**	**% x100**	**Prob. of Exp.>0**	**No.**	**Mean**	**Median**	**Max.**	**Min.**
Lowest	97	0.22	35	0.17	0.66	23	$274	$76	$2,279	$2.00
Low	87	0.19	40	0.20	0.75	30	$273	$118	$2,058	$2.00
Middle	91	0.20	43	0.21	0.77	33	$286	$123	$1,777	$3.00
High	93	0.21	42	0.21	0.79	33	$339	$122	$3,692	$2.00
Highest	81	0.18	43	0.21	0.74	32	$767	$285	$7,596	$1.00
Total	449	1.00	203	1.00	0.74	151	$395	$122	$7,596	$1.00

The severe under-use of care is more significant among fatal injuries and less significant among non-fatal injuries, 45% vs. 88% respectively, see Table C5. Among the non-fatal injuries, the severity of the injury data shows that that probability of care was not alarmingly different raging between 85% to 94%. However, the mean expenditure varied significantly as expected where the low severity injuries (66% of the injuries) spent on average $5 (range: 0.02-2,701) and the high severity injuries (13%) spent on average $90 (range: 0.3-5,917).

Table C5 – Non-Fatal injuries out-of-pocket (OOP) expenditures by injury severity

Severity	Injury Rate	Severity Prob.	Treat Prob.	Exp. Prob.	Total Exp.	Mean Exp. (if Exp.>0)	Max.	Min.
**All**	20,390	1.00	0.88	0.96	$355,437	$21	$5,917	$0.02
**Low**	13,512	0.66	**0.85**	0.96	$54,616	$5	$2,701	$0.02
**Medium**	4,245	0.21	**0.97**	0.98	$85,400	$21	$2,087	$0.03
**High**	2,632	0.13	**0.94**	0.97	$215,761	$90	$5,917	$0.03

Table D1 - Non-fatal injuries analytic decision tree model to estimate total injury cost for a hypothetical population of 100,000 people by injury type

Injury	Injury Rate per 100,000	Prob. of seeking healthcare: Treated	Prob. of seeking healthcare: Not treated	Prob. of expenditure: Cost > 0	Prob. of expenditure: No cost	Branch Total people	Branch Avg. unit cost	Injury total cost	All non-fatal injuries total cost
Fall	7,741	0.92	0.08	0.97	0.03	6,917	$22	$152,180	$355,795
Cut	4,499	0.86	0.14	0.97	0.03	3,743	$9	$33,687	
Blunt object	2,015	0.91	0.09	0.97	0.03	1,786	$18	$32,143	
Transport	1,778	0.96	0.04	0.98	0.02	1,662	$46	$76,434	
Animal bite	1,635	0.84	0.16	0.90	0.10	1,235	$11	$13,583	
Burn	1,068	0.85	0.15	0.95	0.05	857	$11	$9,428	
Drowning	642	0.39	0.61	0.96	0.04	241	$8	$1,930	
Violence	587	0.95	0.05	0.98	0.02	547	$47	$25,726	
Machine	220	0.96	0.04	0.78	0.22	163	$29	$4,739	
Electrocution	146	0.78	0.22	0.94	0.06	107	$38	$4,055	
Suffocation	33	0.62	0.38	0.79	0.21	16	$27	$434	
Poisoning (unintentional)	15	0.87	0.13	0.95	0.05	12	$55	$677	
Suicide attempt	10	0.88	0.12	0.98	0.02	9	$84	$747	
Others	0.3	1.00	0.00	1.00	0.00	0	$93	$32	

Table D2 - Fatal injuries analytic decision tree model to estimate total injury cost for a hypothetical population of 100,000 people by injury type

Injury Rate per 100,000	Prob. of seeking healthcare	Prob. of expenditure	Branch Total people	Injury Avg. unit cost	Injury total cost
Drowning 15	Treated 0.23	Cost > 0 0.59	2	$10	$20
		No cost 0.41			
	Not treated 0.77				
Transport 7	Treated 0.45	Cost > 0 0.72	2	$760	$1,690
		No cost 0.28			
	Not treated 0.55				
Fall 5	Treated 0.86	Cost > 0 0.82	4	$319	$1,146
		No cost 0.18			
	Not treated 0.14				
Suicide 3	Treated 0.38	Cost > 0 0.80	1	$239	$245
		No cost 0.20			
	Not treated 0.62				
Burn 2	Treated 0.96	Cost > 0 0.75	2	$704	$1,084
		No cost 0.25			
	Not treated 0.04				
Violence 2	Treated 0.50	Cost > 0 0.90	1	$324	$249
		No cost 0.10			
	Not treated 0.50				
Electrocution 1	Treated 0.38	Cost > 0 0.40	0	$9	$2
		No cost 0.60			
	Not treated 0.62				

Injury Rate per 100,000	Prob. of seeking healthcare	Prob. of expenditure	Branch Total people	Injury Avg. unit cost	Injury total cost
Suffocation 1	Treated 0.00	Cost > 0 1.00	0	$0	$0
		No cost 0.00			
	Not treated 1.00				
Blunt object 1	Treated 0.56	Cost > 0 0.71	0	$204	$62
		No cost 0.29			
	Not treated 0.44				
Animal bite 1	Treated 0.88	Cost > 0 0.75	0	$328	$147
		No cost 0.25			
	Not treated 0.13				
Poisoning (unintentional) 1	Treated 0.67	Cost > 0 0.80	0	$219	$60
		No cost 0.20			
	Not treated 0.33				
Cut 0	Treated 1.00	Cost > 0 1.00	0	$710	$304
		No cost 0.00			
	Not treated 0.00				
Machine 0	Treated 0.67	Cost > 0 0.74	0	$830	$106
		No cost 0.26			
	Not treated 0.33				

PART 4 – FINANCIAL COPING MECHANISMS

Table E1 - Non-fatal injuries probability of financial distress & coping mechanisms by injury type

External cause of injury	Fall	Cut	Blunt object	Transport	Animal bite	Burn	Near Drowning	Violence	Machine	Electrocu-tion	Suffoca-tion	Poisoning (uninte	Suicide attempt	Other	Total
Total annual non-fatal injuries	90,544	52,622	23,572	20,796	19,126	12,486	7,504	6,860	2,578	1,710	384	174	120	4	**238,480**
No. & portion of non-fatal injuries from individuals	23,836	22,210	11,026	11,594	7,436	2,824	102	2,826	1,910	692	90	48	58	2	**84,654**
reported as source of family income	0.26	0.42	0.47	0.56	0.39	0.23	0.01	0.41	0.74	0.40	0.23	0.28	0.48	0.50	**0.35**
Portion who reported use of any financial cooping mechanism to pay for injury related costs. Data sample is the individuals who were a source of family income	0.97	0.96	0.97	0.97	0.95	0.95	0.86	0.98	0.98	0.96	1.00	1.00	0.97	1.00	**0.97**
Borrow money from Relative/neighbor/friends	0.63	0.73	67.04	0.18	17.82	0.22	0.37	0.11	0.30	0.25	0.41	0.01	0.00	0.00	**0.29**
Clothing and shelter	0.60	0.90	51.88	0.14	21.67	0.35	0.16	0.07	0.25	0.32	0.28	0.01	0.00	0.00	**0.28**
Reduce food	0.29	0.42	29.71	0.07	10.02	0.15	0.06	0.04	0.15	0.14	0.17	0.00	0.00	0.00	**0.14**
Other	0.72	1.04	98.21	0.17	33.91	0.29	0.29	0.07	0.39	0.36	0.90	0.01	0.00	1.00	**0.36**
Took loans from Organization	0.06	0.05	7.00	0.03	1.16	0.02	0.00	0.02	0.04	0.03	0.10	0.00	0.00	0.00	**0.03**
Sell assets	0.02	0.03	3.21	0.01	0.44	0.01	0.00	0.01	0.01	0.00	0.00	0.00	0.00	0.00	**0.01**

Table E2 - Fatal injuries probability of financial distress & coping mechanisms by injury type

External cause of injury	Drowning	Transport	Fall	Suicide	Burn	Violence	Electrocu-tion	Suffoca-tion	Blunt object	Animal bite	Poisoning (uninten-	Cut	Machine	Total
Total annual fatal injuries	172	80	59	39	25	20	13	10	9	8	6	5	3	**449**
No. & portion of non-fatal injuries from individuals	8	47	18	12	3	12	8	1	4	4	2	0	3	**122**
reported as source of family income	0.05	0.59	0.31	0.31	0.12	0.60	0.62	0.10	0.44	0.50	0.33	0.00	1.00	**0.27**
Portion who reported use of any financial cooping mechanism to pay for injury related costs. Data sample is the individuals who were a source of family income	0.75	0.96	1.00	0.83	1.00	0.83	1.00	1.00	1.00	0.75	1.00	0.00	0.67	**0.92**
Borrow money from relative/neighbor/friends	0.50	0.57	0.67	0.33	0.33	0.58	0.38	0.00	0.75	0.75	1.00	0.00	0.67	**0.56**
xpenditure on other basic needs,e.g.clothing and shelter	0.13	0.21	0.44	0.25	0.33	0.25	0.63	0.00	0.00	0.00	0.00	0.00	0.00	**0.25**
Sell assets and household possession	0.13	0.11	0.33	0.25	0.00	0.33	0.25	0.00	0.00	0.25	0.00	0.00	0.00	**0.18**
Reduce family consumption of food	0.25	0.11	0.33	0.08	0.00	0.08	0.13	1.00	0.00	0.00	0.00	0.00	0.00	**0.14**
Took loans from person institution/organization	0.00	0.15	0.11	0.08	0.67	0.17	0.13	0.00	0.00	0.00	0.00	0.00	0.00	**0.12**
Others	0.25	0.13	0.17	0.00	0.67	0.00	0.13	1.00	0.25	0.25	0.00	0.00	0.00	**0.14**

The most common non-exclusive mechanisms among non-fatal injuries were borrowing money from relative/neighbor/friends (29%), reducing consumption of basic goods such as cloths and shelter (28%), reducing food consumption (14%), and other (36%).

Table F1 – Descriptive statistic for variables included in the regression models

Variable	Non-fatal injuries among individuals who were a source of family income (42,327 obs.)		All non-fatal Injuries (119,240 obs.)		All population (1,184,559 obs.)	
	Mean	Std. Dev.	Mean	Std. Dev.	Mean	Std. Dev.
Reported financial distress (using at least one coping mechanism)	0.97	0.18	-	-	-	-
Injury: Fall	0.28	0.45	0.38	0.49	0.04	0.19
Injury: Cut	0.26	0.44	0.22	0.41	0.02	0.15
Injury: Transport	0.14	0.34	0.09	0.28	0.01	0.09
Injury: Blunt object	0.13	0.34	0.10	0.30	0.01	0.10
Injury: Animal Bite	0.09	0.28	0.08	0.27	0.01	0.09
Injury: Violence	0.03	0.18	0.03	0.17	0.00	0.05
Injury: Burn	0.03	0.18	0.05	0.22	0.01	0.07
Injury: Machine	0.02	0.15	0.01	0.10	0.00	0.03
Injury: Electrocution	0.01	0.09	0.01	0.08	0.00	0.03
Injury: Drowning	0.001	0.035	0.031	0.175	0.003	0.057
Injury: Suffocation	0.001	0.033	0.002	0.040	0.000	0.013
Injury: Suicide attempt	0.001	0.026	0.001	0.022	0.000	0.009
Injury: Poisoning (unintentional)	0.001	0.024	0.001	0.027	0.000	0.009
Injury severity: Low	0.64	0.48	0.67	0.47	0.07	0.25
Injury severity: Medium	0.23	0.42	0.20	0.40	0.02	0.14
Injury severity: High	0.12	0.33	0.12	0.33	0.01	0.11
Family/household size	5.15	2.18	5.36	2.24	5.15	2.03
Female	0.21	0.40	0.44	0.50	0.51	0.50
Secondary Education	0.28	0.45	0.26	0.44	0.30	0.46
District: Chandpur	0.46	0.50	0.55	0.50	0.52	0.50
District: Comilla	0.03	0.18	0.04	0.19	0.02	0.15
District: Sirajgonj	0.17	0.38	0.13	0.34	0.09	0.29
District: Sherpur	0.16	0.37	0.14	0.35	0.19	0.40
District: Narshindi	0.17	0.38	0.14	0.35	0.17	0.38
Age less than1 yr. old	0.00	0.02	0.00	0.07	0.02	0.14
Age 1 to 4 yrs. Old	0.00	0.06	0.09	0.28	0.08	0.27
Age 5 to 14 yrs. old	0.02	0.15	0.20	0.40	0.24	0.43
Age 15 to 24 yrs. old	0.10	0.31	0.13	0.33	0.17	0.37
Age 25 to 64 yrs. old	0.78	0.41	0.52	0.50	0.44	0.50
Age 64 yrs. old and more	0.09	0.28	0.07	0.25	0.06	0.24
SES Highest	0.16	0.37	0.19	0.39	0.22	0.41
SES High	0.20	0.40	0.21	0.41	0.21	0.41
SES Medium	0.21	0.40	0.21	0.41	0.20	0.40
SES Low	0.21	0.41	0.20	0.40	0.19	0.39
SES Lowest	0.22	0.41	0.19	0.40	0.18	0.39

Financial coping mechanism data was not collected for injuries from individuals who were not reported as a source of family income. Thus, the regression analysis included all the 42,327 non-fatal injuries reported during the six months prior to the household survey but only from the individuals who were reported being a source of family income. A fatal injuries regression analysis was not done or included because the number of injuries was too small to draw statistically significant results for that group. The table below shows the full list of regression analysis models tested. The regressions are interpreted as the percent change in the probability of financial distress given each injury compared to the base case injury cases. In this case, the base case injuries are the four injuries with the lowest injury rate and which make less than 1% of the total injury burden. The regression controls for demographic and SES factors in order to show the association between financial distress and the most common injuries.

Table F2 – Probit regression models

	1	2	2	3	4	5	6	7	8	9	10	11	12	13	14	15	16	17	18	19	20	21
**VARIABLES**	**Fin. Stress**	**Fin. Stress**	**Fin. Stress**	**Fin. Stress**	**Fin. Stress**	**Fin. Stress**	**Fin. Stress**	**Fin. Stress**	**Fin. Stress**	**Fin. Stress**	**Fin. Stress**	**Fin. Stress**	**Fin. Stress**	**Fin. Stress**	**Fin. Stress**	**Fin. Stress**	**Fin. Stress**	**Fin. Stress**	**Fin. Stress**	**Fin. Stress**	**Fin. Stress**	**Fin. Stress**
Injury: Transport	0.00682***	0.00746***	0.0115***	0.0122***	0.0106***	0.0123***	0.00362	0.0228*	0.0186	0.0199	0.0210*	0.0174	0.0168	0.0170	0.0161	0.0151	0.0159	0.0165	0.0174	0.0173	0.0173	0.0177
	[0.00264]	[0.00264]	[0.00270]	[0.00289]	[0.00295]	[0.00299]	[0.00342]	[0.0123]	[0.0122]	[0.0122]	[0.0121]	[0.0121]	[0.0121]	[0.0121]	[0.0121]	[0.0119]	[0.0120]	[0.0120]	[0.0120]	[0.0120]	[0.0120]	[0.0119]
Injury: Violence		0.0221***	0.0261***	0.0267***	0.0252***	0.0268***	0.0181***	0.0374***	0.0350***	0.0325**	0.0333**	0.0312**	0.0312**	0.0313**	0.0303**	0.0290**	0.0266**	0.0273**	0.0286**	0.0286**	0.0285**	0.0284**
		[0.00604]	[0.00605]	[0.00614]	[0.00617]	[0.00618]	[0.00638]	[0.0134]	[0.0133]	[0.0134]	[0.0133]	[0.0132]	[0.0132]	[0.0133]	[0.0132]	[0.0131]	[0.0132]	[0.0132]	[0.0132]	[0.0132]	[0.0131]	[0.0131]
Injury: Fall			0.0133***	0.0140***	0.0125***	0.0141***	0.00548*	0.0247**	0.0220*	0.0244**	0.0263**	0.0266**	0.0264**	0.0262**	0.0258**	0.0243**	0.0164	0.0171	0.0179	0.0179	0.0179	0.0182
			[0.00208]	[0.00232]	[0.00239]	[0.00245]	[0.00295]	[0.0122]	[0.0121]	[0.0121]	[0.0120]	[0.0120]	[0.0120]	[0.0120]	[0.0120]	[0.0118]	[0.0119]	[0.0119]	[0.0119]	[0.0119]	[0.0118]	[0.0118]
Injury: Cut				0.00145	-0.000105	0.00157	-0.00705**	0.0122	0.0101	0.0152	0.0167	0.0152	0.0152	0.0152	0.0145	0.0136	0.0155	0.0166	0.0178	0.0177	0.0176	0.0180
				[0.00218]	[0.00226]	[0.00232]	[0.00286]	[0.0122]	[0.0120]	[0.0121]	[0.0120]	[0.0120]	[0.0119]	[0.0120]	[0.0119]	[0.0118]	[0.0119]	[0.0118]	[0.0118]	[0.0118]	[0.0118]	[0.0118]
Injury: Burn					-0.0117***	-0.0100**	-0.0186***	0.000612	-0.000612	0.00454	0.00653	0.0108	0.0103	0.0107	0.00995	0.00912	0.0127	0.0142	0.0154	0.0154	0.0153	0.0157
					[0.00429]	[0.00431]	[0.00462]	[0.0127]	[0.0126]	[0.0126]	[0.0125]	[0.0125]	[0.0125]	[0.0125]	[0.0125]	[0.0124]	[0.0124]	[0.0124]	[0.0124]	[0.0124]	[0.0123]	[0.0123]
Injury: Machine						0.0278***	0.0192**	0.0384***	0.0348**	0.0376***	0.0380***	0.0346**	0.0342**	0.0350**	0.0341**	0.0333**	0.0316**	0.0325**	0.0327**	0.0326**	0.0324**	0.0330**
						[0.00751]	[0.00767]	[0.0141]	[0.0140]	[0.0140]	[0.0139]	[0.0139]	[0.0139]	[0.0139]	[0.0139]	[0.0138]	[0.0139]	[0.0139]	[0.0138]	[0.0138]	[0.0138]	[0.0138]
Injury: Electrocution						-0.00352	-0.0121	0.00711	0.00692	0.00776	0.00842	0.00802	0.00726	0.00737	0.00656	0.00568	0.00817	0.00875	0.00916	0.00903	0.00923	0.0103
						[0.00867]	[0.00882]	[0.0147]	[0.0146]	[0.0146]	[0.0145]	[0.0144]	[0.0144]	[0.0144]	[0.0144]	[0.0143]	[0.0143]	[0.0143]	[0.0143]	[0.0143]	[0.0143]	[0.0143]
Injury: Animal Bite							-0.0190***	0.000229	9.57e-05	0.00489	0.00632	0.00600	0.00600	0.00581	0.00513	0.00398	0.00893	0.00926	0.00941	0.00938	0.00922	0.00954
							[0.00342]	[0.0123]	[0.0122]	[0.0122]	[0.0121]	[0.0121]	[0.0121]	[0.0121]	[0.0121]	[0.0119]	[0.0120]	[0.0120]	[0.0120]	[0.0119]	[0.0119]	[0.0119]
Injury: Blunt object								0.0199	0.0172	0.0211*	0.0221*	0.0193	0.0190	0.0193	0.0184	0.0174	0.0111	0.0121	0.0128	0.0128	0.0128	0.0132
								[0.0123]	[0.0122]	[0.0122]	[0.0121]	[0.0121]	[0.0121]	[0.0121]	[0.0121]	[0.0119]	[0.0120]	[0.0120]	[0.0120]	[0.0120]	[0.0120]	[0.0119]
Injury severity: Medium									0.0286***	0.0313***	0.0310***	0.0301***	0.0301***	0.0301***	0.0302***	0.0301***	0.0214***	0.0214***	0.0218***	0.0218***	0.0218***	0.0218***
									[0.00260]	[0.00262]	[0.00261]	[0.00260]	[0.00260]	[0.00260]	[0.00260]	[0.00260]	[0.00259]	[0.00259]	[0.00259]	[0.00259]	[0.00259]	[0.00259]
Injury severity: High										0.0218***	0.0218***	0.0211***	0.0212***	0.0211***	0.0211***	0.0211***	0.0154***	0.0158***	0.0163***	0.0163***	0.0164***	0.0164***
										[0.00322]	[0.00322]	[0.00322]	[0.00322]	[0.00322]	[0.00322]	[0.00321]	[0.00322]	[0.00322]	[0.00322]	[0.00322]	[0.00322]	[0.00322]
Family/household size											0.00288***	0.00251***	0.00244***	0.00245***	0.00239***	0.00246***	0.00104***	0.000857**	0.000839**	0.000833**	0.000831**	0.00105**
											[0.000418]	[0.000413]	[0.000413]	[0.000412]	[0.000409]	[0.000416]	[0.000398]	[0.000401]	[0.000402]	[0.000401]	[0.000400]	[0.000412]
Female												-0.0137***	-0.0136***	-0.0137***	-0.0138***	-0.0136***	-0.00539***	-0.00615***	-0.00588***	-0.00585***	-0.00567***	-0.00569***
												[0.00206]	[0.00206]	[0.00206]	[0.00206]	[0.00205]	[0.00200]	[0.00201]	[0.00202]	[0.00202]	[0.00202]	[0.00201]
Secondary education													0.00487**	0.00557***	0.00523***	0.00525***	-9.52e-05	-0.000693	0.000274	0.000353	0.000908	0.00235
													[0.00199]	[0.00201]	[0.00201]	[0.00201]	[0.00202]	[0.00203]	[0.00205]	[0.00205]	[0.00206]	[0.00213]
Age: 15_24yr														-0.00676**	-0.00193	0.0112**	0.00564	0.00583	0.00548	0.00550	0.00531	0.00498
														[0.00274]	[0.00358]	[0.00501]	[0.00487]	[0.00486]	[0.00487]	[0.00487]	[0.00486]	[0.00487]
Age: 25_64yr															0.00544**	0.0187***	0.0104**	0.0106**	0.0102**	0.0102**	0.0102**	0.0103**
															[0.00261]	[0.00441]	[0.00425]	[0.00424]	[0.00425]	[0.00425]	[0.00425]	[0.00425]
Age: 65up																0.0187***	0.0128**	0.0129**	0.0127**	0.0129**	0.0131***	0.0132***
																[0.00523]	[0.00506]	[0.00505]	[0.00505]	[0.00505]	[0.00505]	[0.00505]
District: Chandpur																	0.0596***	0.0574***	0.0526***	0.0525***	0.0527***	0.0529***
																	[0.00277]	[0.00283]	[0.00298]	[0.00298]	[0.00298]	[0.00298]
District: Sherpur																		-0.00681***	-0.0114***	-0.0115***	-0.0111***	-0.0111***
																		[0.00199]	[0.00227]	[0.00227]	[0.00227]	[0.00227]
District: Narshindi																			-0.00962***	-0.00960***	-0.00924***	-0.00865***
																			[0.00224]	[0.00224]	[0.00225]	[0.00226]
SES: Med																				0.00232	0.00364	0.00609**
																				[0.00219]	[0.00225]	[0.00245]
SES: Lw																					0.00507**	0.00765***
																					[0.00219]	[0.00241]
SES: Lst																						0.00600**
																						[0.00238]
Observations	42,327	42,327	42,327	42,327	42,327	42,327	42,327	42,327	42,327	42,327	42,327	42,327	42,327	42,327	42,327	42,327	42,327	42,327	42,327	42,327	42,327	42,327

Standard errors in brackets. *** p<0.01, ** p<0.05, * p<0. Red colored text are coefficients that are statistically significant and blue filled cells are coefficients with a negative sign.
